# Using a classification model for determining the value of liver radiological reports of patients with colorectal cancer

**DOI:** 10.3389/fonc.2022.913806

**Published:** 2022-11-21

**Authors:** Wenjuan Liu, Xi Zhang, Han Lv, Jia Li, Yawen Liu, Zhenghan Yang, Xutao Weng, Yucong Lin, Hong Song, Zhenchang Wang

**Affiliations:** ^1^ Department of Radiology, Beijing Friendship Hospital, Capital Medical University, Beijing, China; ^2^ School of Computer Science and Technology, Beijing Institute of Technology, Beijing, China; ^3^ School of Biological Science and Medical Engineering, Beihang University, Beijing, China

**Keywords:** natural language processing, colorectal cancer, liver lesion, medical imaging report, classification model

## Abstract

**Background:**

Medical imaging is critical in clinical practice, and high value radiological reports can positively assist clinicians. However, there is a lack of methods for determining the value of reports.

**Objective:**

The purpose of this study was to establish an ensemble learning classification model using natural language processing (NLP) applied to the Chinese free text of radiological reports to determine their value for liver lesion detection in patients with colorectal cancer (CRC).

**Methods:**

Radiological reports of upper abdominal computed tomography (CT) and magnetic resonance imaging (MRI) were divided into five categories according to the results of liver lesion detection in patients with CRC. The NLP methods including word segmentation, stop word removal, and n-gram language model establishment were applied for each dataset. Then, a word-bag model was built, high-frequency words were selected as features, and an ensemble learning classification model was constructed. Several machine learning methods were applied, including logistic regression (LR), random forest (RF), and so on. We compared the accuracy between priori choosing pertinent word strings and our machine language methodologies.

**Results:**

The dataset of 2790 patients included CT without contrast (10.2%), CT with/without contrast (73.3%), MRI without contrast (1.8%), and MRI with/without contrast (14.6%). The ensemble learning classification model determined the value of reports effectively, reaching 95.91% in the CT with/without contrast dataset using XGBoost. The logistic regression, random forest, and support vector machine also achieved good classification accuracy, reaching 95.89%, 95.04%, and 95.00% respectively. The results of XGBoost were visualized using a confusion matrix. The numbers of errors in categories I, II and V were very small. ELI5 was used to select important words for each category. Words such as “no abnormality”, “suggest”, “fatty liver”, and “transfer” showed a relatively large degree of positive correlation with classification accuracy. The accuracy based on string pattern search method model was lower than that of machine learning.

**Conclusions:**

The learning classification model based on NLP was an effective tool for determining the value of radiological reports focused on liver lesions. The study made it possible to analyze the value of medical imaging examinations on a large scale.

## Introduction

Liver metastasis occurs in approximately 30% of patients with colorectal cancer (CRC), and is the cause of death in around two thirds of the death from CRC (1). Medical imaging plays a great part in its diagnosis, with the common examination methods including ultrasound, computed tomography (CT), and magnetic resonance imaging (MRI). Patients with CRC diagnosed by enteroscopy and pathology typically undergo liver medical imaging to screen for metastasis, compared with the common purposes for upper abdominal imaging, those patients may prompt an urgent need for efficient detection of suspect lesions of metastasis, rather than a detailed description of all normal organs. On the other hand, the patterns of liver metastasis remain to be further discovered by means of medical meta-data, free-text radiology reports contain highly informative diagnostic messages nevertheless require to be processed by NLP techniques before converted into a useable dataset, so far there is a lack of methods to hierarchically classify the certainty of radiology reports.

Moreover, as liver lesions could relate to multiple diseases and presented on fibrosis background, resulting in the ambiguity and hedging results in radiology, however, there is scarcely. In this study, liver radiological reports of patients with CRC were divided into five categories according to the radiologist’s opinion. Category I was defined as liver without any abnormality; I was defined as a small liver lesion without clinical significance with no clinical recommendations made; III was defined as clinically significant liver lesions accidentally discovered and unrelated to CRC; IV was defined as suspected liver metastasis needing further clinical examination or follow-up observation; V was defined as positive for liver metastasis. Once the model was able to accurately classify the findings in radiological reports, clinicians could intuitively obtain results and be provided with decision support for further clinical management of CRC patients.

Recent studies have demonstrated the feasibility of natural language processing (NLP) methods in extracting information from radiology reports, helping to overcome the obstacles faced when reusing medical imaging report information in clinical research and other medical and health care applications (2, 3). Some recent studies have also used statistical NLP methods to make differential diagnoses. Tong et al. (4) used random forest (RF) and convolutional neural network approaches to identify disease entities and established a disease classification model for ulcerative colitis, Crohn’s disease, and intestinal tuberculosis. Eskin et al. (5) used a support vector machine (SVM) algorithm and gene sequence kernel to predict the position of protein in cytoplasm, achieving 87% precision and 71% recall. Al-Garadi et al. (6) proposed a bidirectional encoder representation from a transformers based model, a fusion learning model, and bi-directional long short-term memory based model, and then used these models to detect self-reports of prescription medication abuse on Twitter. Brown et al. (7) used three machine learning models, namely logistic regression (LR), SVM, and RF, to predict future use of radiology department resources.

Because the structured report template may sometimes not fully express the ideas of radiologists, liver imaging reports are still written in free text in most hospitals in China. The complexity of Chinese language, the writting style and template of radiology reports have significant differences due to the perference across different hospitals, a comprehensive analysis of semantic features in radiology reports is quite attractive but hard to complete in short time. Therefore, the rule-based NLP method was used to extract information from imaging reports written by specific templates in Chinese, just such as breast cancer (8, 9). The NLP model based on machine learning is used in more researches on imaging reports in Chinese, and good results have been achieved (10, 11).

An ensemble learning classification model of liver medical imaging reports of patients with CRC should be sensitive to the relationships between different examination methods and their clinical significance. Based on these motivations, the purpose of this study was to establish an ensemble learning classification model based on NLP methods to classify the of radiological reports concerning liver lesion detection in patients with CRC and written in Chinese free text. Such a classification model could improve the efficiency of clinicians’ interpretations of medical imaging examination results, and make it possible to analyze the value of medical imaging examinations on a large scale.

## Methods

### Data set and data preparation

**Figure 1:**
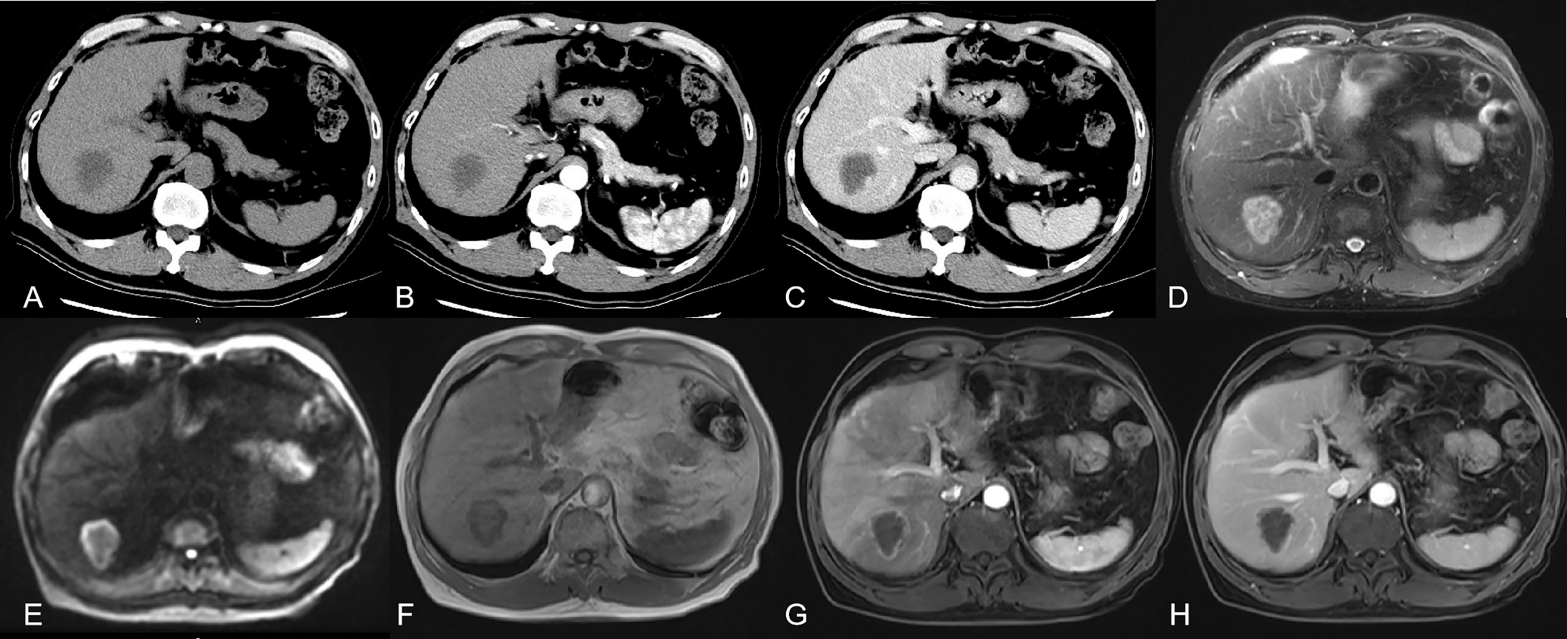
showed a 66 year old male patient with rectal cancer. **(A)** CT without contrast showed there was an irregular low-density focus in S7 segment of the liver, which could not be well diagnosed qualitatively. **(B, C)** CT with contrast showed there seemed to be slight enhancement at the edge of the lesion. CT imaging combined with the patient’s history could make an imaging diagnosis of suspected liver metastasis. **(D-F)** MRI without contrast could basically characterize the lesion as malignant. **(G, H)** with contrast could make a definite diagnosis of metastases.

This study focused on CT/MRI examinations of the upper abdomen of patients with CRC that were performed at our institution between October 1, 2014 and April 30, 2021. The medical imaging reports on the liver were extracted from the medical imaging information system of a clinical medicine big data platform. The dataset included examination methods and the text of the medical imaging reports. Examination methods included CT without contrast, CT with/without contrast, MRI without contrast and MRI with/without contrast (**Figure 1**). A radiological report usually consists of two parts: image description and diagnostic conclusion. To generate features from each report, we focused on the conclusion section of the report. For the purposes of this classification problem, the conclusion section was considered the highest yielding portion in respect to the clinical significance of the report, because it could include information such as specific diagnosis, differential diagnosis, or recommendations for follow-up diagnostic studies. Cases were excluded according to the following criteria (1): follow-up reports, (2) covered liver surgery, (3) incomplete reports.

In preparation for the model development, all liver reports were classified as I to V according to their clinical significance. Reports were reviewed and manually labeled by 2 clinicians with at least 3 years of experience reviewing upper abdominal radiology reports, the labeling criteria was made previously at the consensus of all authors. The classification and labeling of a radiologist served as the reference standard. The code of patient, imaging examination method, imaging report and manual label of clinical significance were shown in **eTable 1** in Supplement.

### Problem definition

**Figure 2:**
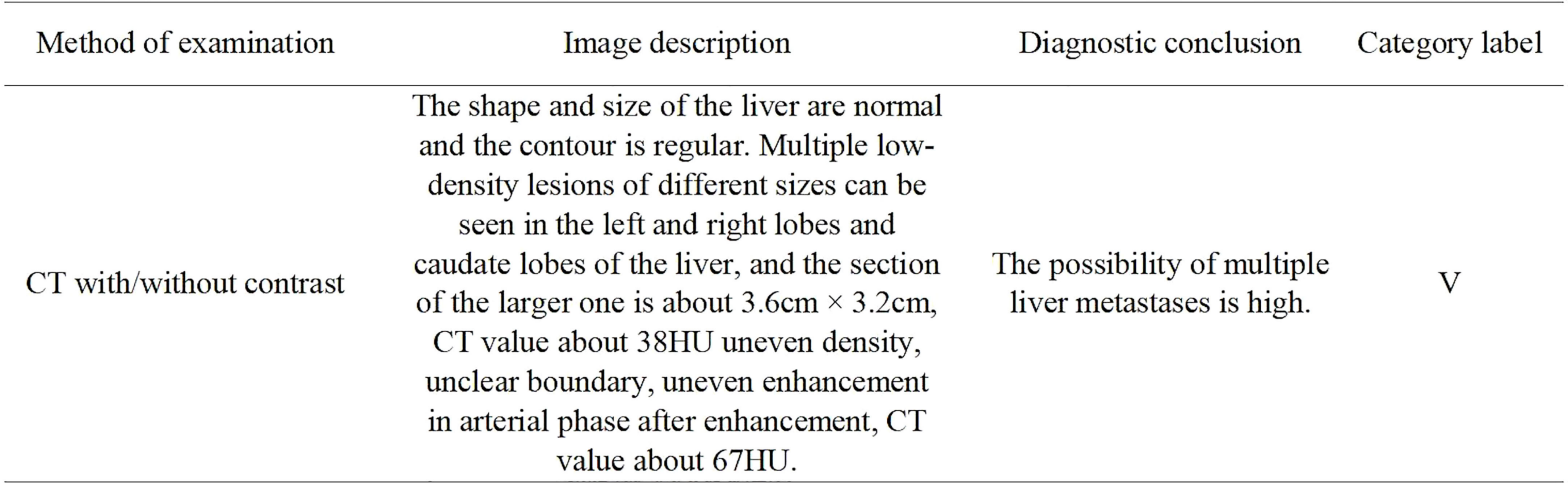
A representative original liver CT with/without contrast report and category label of patient with colorectal cancer. The report consists of an imaging description and diagnostic conclusion. For publication purposes, we provided English version of Chinese words from the imaging reports.

This study defined the task of determining the clinical significance of liver reports of a population with CRC as a multi-classification problem. The results of the liver medical imaging examinations were classified into five categories. Category I was defined as normal liver without any abnormality; II was defined as a small liver lesion without clinical significance and without clinical recommendations; III was defined as clinically significant liver lesions of unknown nature, with the radiologist putting forward clinical suggestions; IV was defined as suspected liver metastasis in need of further clinical examination or follow-up observation; and V was defined as positive for liver metastasis. Categories I and II generally excluded liver metastasis of CRC, while category V was a positive diagnosis of liver metastasis. Categories III and IV required special attention from clinicians. A representative original liver report and it’s category label were shown in **Figure 2**.

### Data processing

Before classification, NLP was used to extract language features. First, using the Python package “jieba”, Chinese word segmentation was applied to the description to tokenize the input text. Second, a stop word dictionary was established to remove stop words. The stop words mainly included adjectives and some adverbs and connectives. Currently, there are approximately 2000 stop words in the dictionary.

The core of the text classification involved the extraction of key features reflecting the characteristics of the text and capturing the mapping between the features and the categories. Regardless of the frequency of a word, as long as it appeared, it would be marked with 1 in the corresponding position, otherwise it would be marked with 0. Under the general belief that the more a word appeared in a text the more important it was, and therefore the greater the weight it had. We built a bag-of-words model, selected words with a word frequency of more than 1000 as features, and used 1-gram and 1–3-gram methods. Then, non-participles were also directly tried as features.

### Development of classifiers

We studied the descriptions of the liver and the conclusions of all medical imaging reports, and applied 1-gram and 1–3-gram language model methods. The validation set was then randomly divided into proportions of 1:5, and 5-fold cross-validation was performed. Details are as follows.

We group the original data sets. One part is used as the training set to train the model, and the other part is used as the test set to evaluate the model. The 5-fold cross validation reduces the variance by averaging the training results of 5 different groups, so the performance of the model is less sensitive to the division of data. (1)The first step is to randomly divide the original data into 5 copies without repeated sampling. (2) The second step is to select one of them as the test set each time, and the remaining four as the training set for model training. (3) The third step is to repeat the second step for 5 times, so that each subset has one chance as the test set and the rest as the training set. After training on each training set, a model is obtained, Use this model to test on the corresponding test set, calculate and save the evaluation indicators of the model. (4) The fourth step is to calculate the average value of the five groups of test results as the estimation of the model accuracy and as the performance index of the model under the current 5-fold cross validation.

The value of the examination results itself was the classification problem, and therefore general pattern classification methods could be used for text research, and the following methods were applied: LR, RF, multinomial naive Bayes (NB), multi-layer perceptron (MLP), k-nearest neighbor algorithm (KNN), SVM, and extreme gradient boosting (XGBoost).

LR is a classification and prediction algorithm (12) that can predict the probability of future results based on the performance of historical data. NB models assume that the features are generated by a simple polynomial distribution (13), and multinomial NB is usually used for text classification. Its features refer to the numbers or frequency of occurrences of words in the text being classified. The MLP is a feed forward artificial neural network model that maps multiple input datasets to a single output dataset (14). When a KNN algorithm is given a training dataset, for a new input instance it finds the K instances closest to that instance in the training dataset, with most of these K instances belonging to a certain class, and then assigns the input instance into this category (15). The basic SVM model finds the best separation hyperplane in feature space to maximize the interval between positive and negative samples in the training set (16).

RF and XGBoost are both ensemble learning methods. The idea of ensemble learning is to solve the inherent shortcomings of a single model or a certain set of parameter models, so as to integrate more models, learn from each other’s strengths, and avoid limitations. RF is the product of the idea of ensemble learning, and integrates many decision trees into a forest, where together they are used to predict the final result (17). XGBoost is an efficient implementation of the gradient boosting decision tree (18). The base learner in XGBoost can be either classification and regression trees (gbtree), or a linear (gblinear) classifier.

We adopted a string pattern research method to further explore the efficacy of using key-word based technique to identify critical information and properly classify radiology reports. Two groups of string patterns are developed, group 1 contains key words representing clinical tendency, addressed by 3 experienced radiologists according to their expertise. Group 2 applies a vectorization strategy: Term Frequency-Inverse Document frequency (TF-IDF), a class-specified lexicon is built to segment the sentences, then a vector is created through the statistics of segmented words to represent a text body. The equation for TF-DF is shown in Eqs.1

For a term i in document j:


$${\text{w}}_{\text{i,j}}=tf_{i,j}\times \text{log}\left(\frac{\text{N}}{\text{d}f_{i}}\right)$$

*tf_i,j_* = number of occurrences of *i* in *j*

d*f_i_* = number of documents containing *i*

N=total number of documents

**Eqs.1 Equation of TF-IDF Embedding**

**Figure 3:**
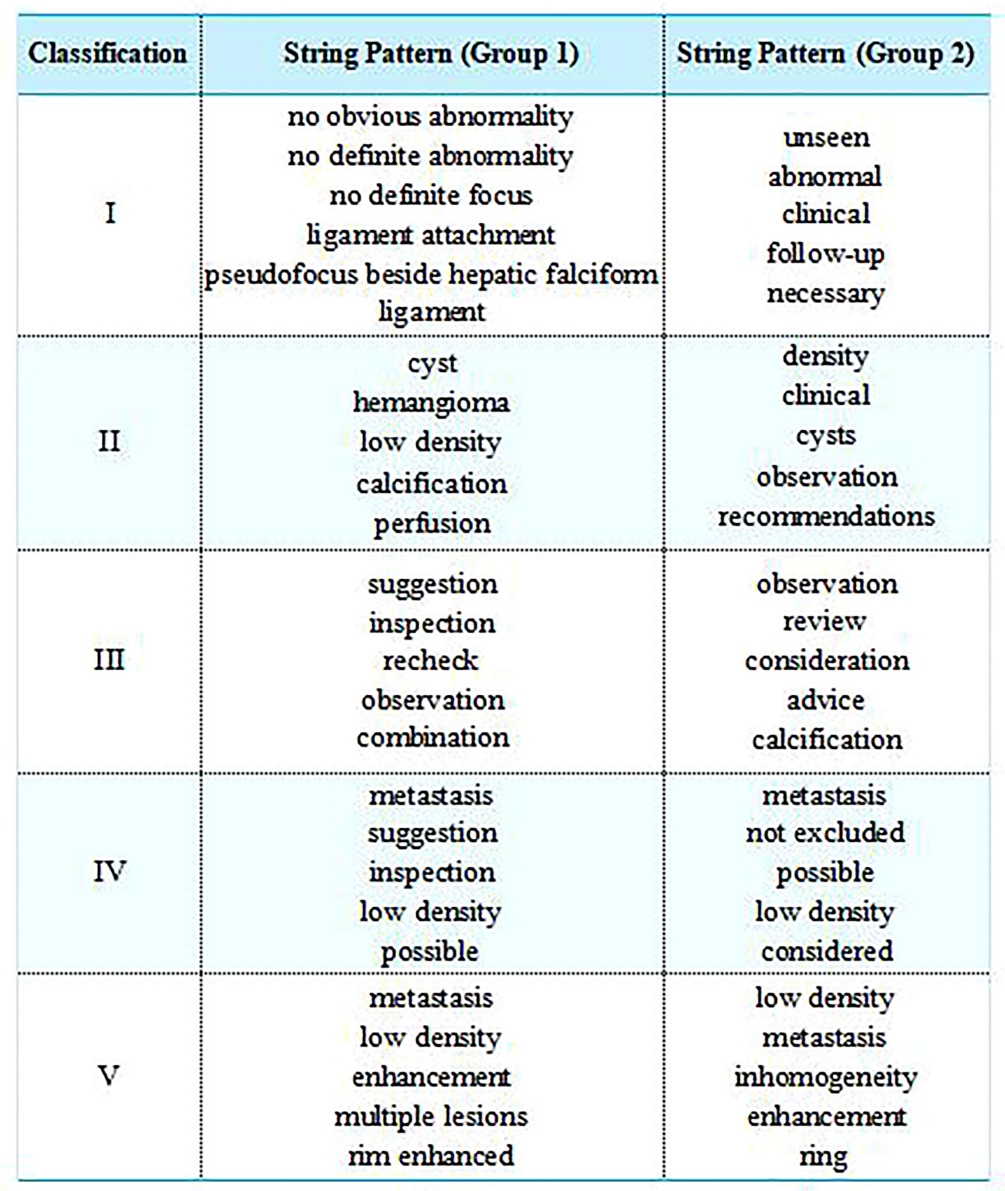
The string patterns defined by 3 experienced radiologists (Group I) and calculated by TF-IDF method (Group2). For publication purposes, we provided English version of Chinese words from the imaging reports.

The string patterns defined by 3 experienced radiologists (Group I) and calculated by TF-IDF method (Group2) were shown in **Figure 3**.

### Ethics approval and consent to participate

As the data were retrospectively collected for administrative purposes and completely anonymized, this study does not fall within the scope of the Medical Research Involving Human Subjects Act (19). Accordingly, the study obtained permission to use anonymized data and a full waiver for the requirement for informed patient consent from the Medical Ethics Review Board of Beijing Friendship Hospital Affiliated to Capital Medical University (reference number 2021-P2-144-01).

## Results

### Basic characteristics of the study population

We acquired a total of 2790 reports, with 10.2% covering CT without contrast, 73.3% CT with/without contrast, 1.8% MRI without contrast, and 14.6% MRI with/without contrast. **Table 1** summarizes the numbers and proportions of the four methods in each of the radiologist’s five classifications. In category V, the lowest proportion of examinations was CT without contrast, whereas the highest proportion of examinations was MRI with/without contrast performed for the purpose of detecting liver metastasis.

**Table 1:** Dataset in each diagnosis category according to the different examination methods.

Method of examination	Number of reports (proportion)					
	Total	Ⅰ	Ⅱ	III	IV	Ⅴ
CT without contrast	286 (100%)	105 (36.7%)	57 (19.9%)	99 (34.6%)	22 (7.7%)	3 (1.0%)
CT with/without contrast	2047 (100%)	602 (29.4%)	546 (26.7%)	639 (31.2%)	144 (7.0%)	116 (5.7%)
MRI without contrast	49 (100%)	7 (14.3%)	12 (24.5%)	16 (32.7%)	8 (16.3%)	6 (12.2%)
MRI with/without contrast	408 (100%)	16 (3.9%)	90 (22.1%)	129 (31.6%)	66 (16.2%)	107 (26.2%)

CT, computed tomography; MRI, magnetic resonance imaging.

This study used LR, RF, multinomial NB, MLP, KNN, SVM, and XGBoost to classify the examination results. The results of the 1-gram and 1–3-gram models are shown in **Tables 2**, **3**, respectively. The accuracy of XGBoost was the highest, reaching 95.91%.

**Table 2:** The result of 5-fold cross-validation with 1 gram language model.

	CV 1	CV 2	CV 3	CV 4	CV 5	Average
LR	0.9618	0.9574	0.9579	0.9594	0.9579	0.9589
RF	0.9486	0.9545	0.9496	0.9481	0.9491	0.9500
Multinomial NB	0.9330	0.9359	0.9354	0.9290	0.9310	0.9329
MLP	0.9442	0.9437	0.9413	0.9364	0.9422	0.9416
KNN	0.8694	0.8733	0.8753	0.8723	0.8679	0.8716
SVM	0.9515	0.9511	0.9491	0.9491	0.9511	0.9504
XGBoost	0.9589	0.9579	0.9603	0.9594	0.9589	0.9591

CV, cross-validation; LR, logistic regression; RF, random forest; NB, naive Bayes; MLP, multi-layer perceptron; KNN, k-nearest neighbor; SVM, support vector machine; XGBoost, extreme gradient boosting.

**Table 3:** The results of 5-fold cross-validations with the 1–3-gram language model.

	CV 1	CV 2	CV 3	CV 4	CV 5	Average
LR	0.9545	0.9535	0.9525	0.9540	0.9540	0.9537
RF	0.9403	0.9457	0.9452	0.9437	0.9374	0.9424
Multinomial NB	0.9183	0.9202	0.9207	0.9154	0.9168	0.9183
MLP	0.9310	0.9300	0.9344	0.9315	0.9281	0.9310
KNN	0.8127	0.8112	0.8136	0.8078	0.8083	0.8107
SVM	0.9310	0.9315	0.9339	0.9334	0.9325	0.9325
XGBoost	0.9618	0.9569	0.9574	0.9584	0.9603	0.9590

CV, cross-validation; LR, logistic regression; RF, random forest; NB, naive Bayes; MLP, multi-layer perceptron; KNN, k-nearest neighbor; SVM, support vector machine; XGBoost, extreme gradient boosting.

The accuracy of the string model based on expert experience (group 1) is 83.78%, and TF-IDF model (group 2) is 64.95%. The accuracy based on string pattern search method model is lower than machine learning (**Table 4**).

**Table 4:** The comparation of results based on machine learning model and string pattern search method.

	ML							SP	
	LR	RF	Multinomial NB	MLP	KNN	SVM	XGBoost	Group 1	Group 2
Accuracy	0.9589	0.9500	0.9329	0.9416	0.8716	0.9504	0.9591	0.8378	0.6495

ML, machine learning; SP, String Pattern; LR, logistic regression; RF, random forest; NB , naive Bayes; MLP, multi-layer perceptron; KNN, k-nearest neighbor; SVM, support vector machine; XGBoost, extreme gradient boosting.

### Visualization of the classifications

**Figure 4:**
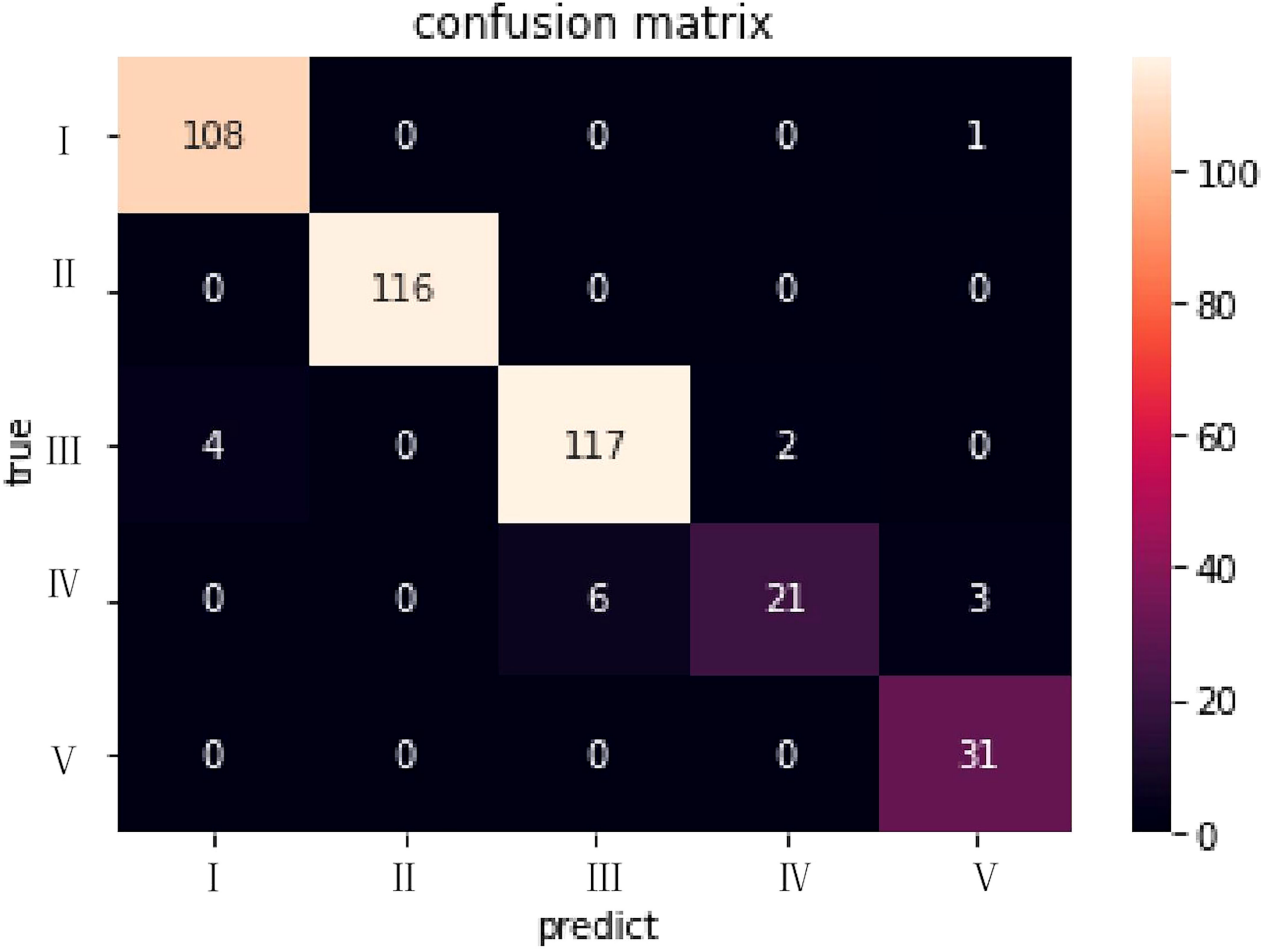
The confusion matrix for the XGBoost model in the dataset of CT with/without contrast. The abscissa represented the predicted results and the ordinate the actual results.

To present the classification results more intuitively, we visualized the results using the ELI5 algorithm (20). A confusion matrix of XGBoost results with 5-fold cross-validation classification is shown in **Figure 4**. The abscissa represented the predicted results and the ordinate the actual results. The numbers of errors in categories I, II and V were very small. The results of these reports were usually positive and easily classified, and were consistent with the radiologist.

**Figure 5:**
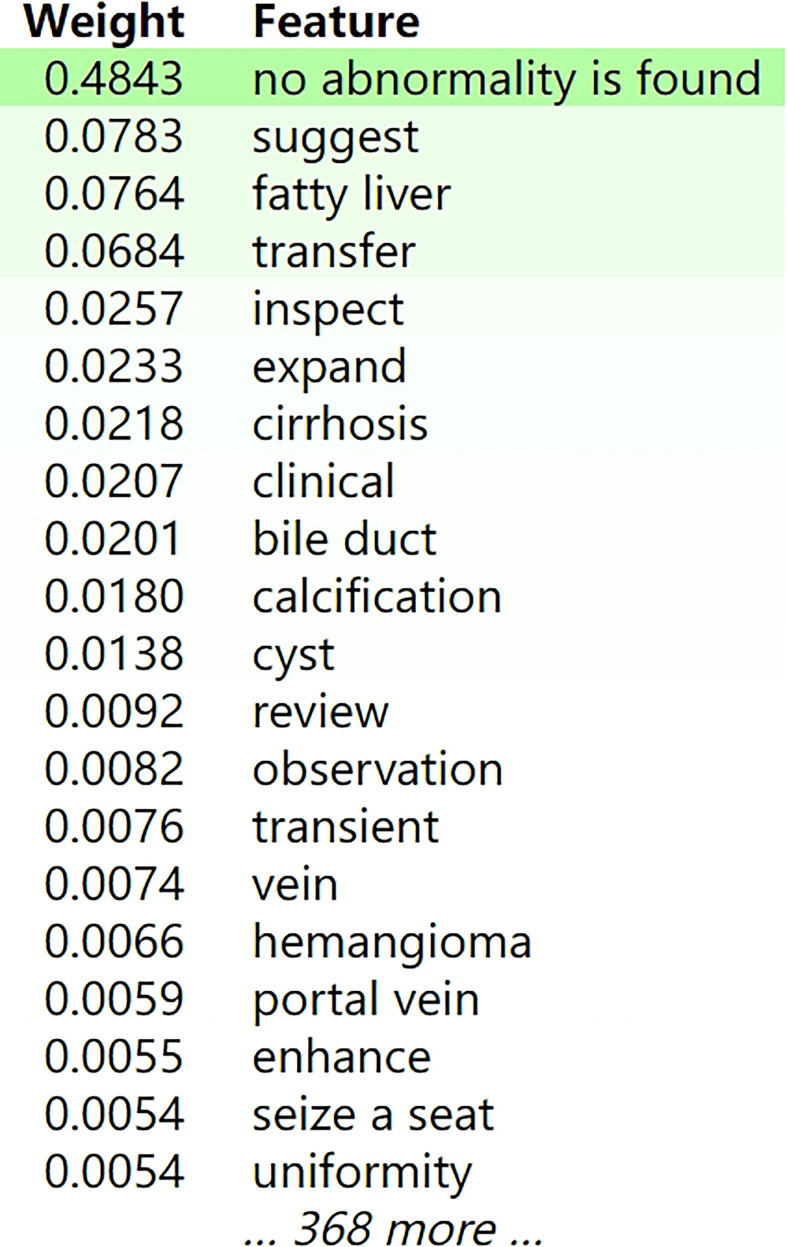
The feature weights of the XGBoost model for the general classification accuracy. Green represented positive correlation, and the weight represented the contribution. The darker the green, the stronger the correlation between the word and classification. For publication purposes, we provided English version of Chinese words from the imaging reports.

**Figure 6:**
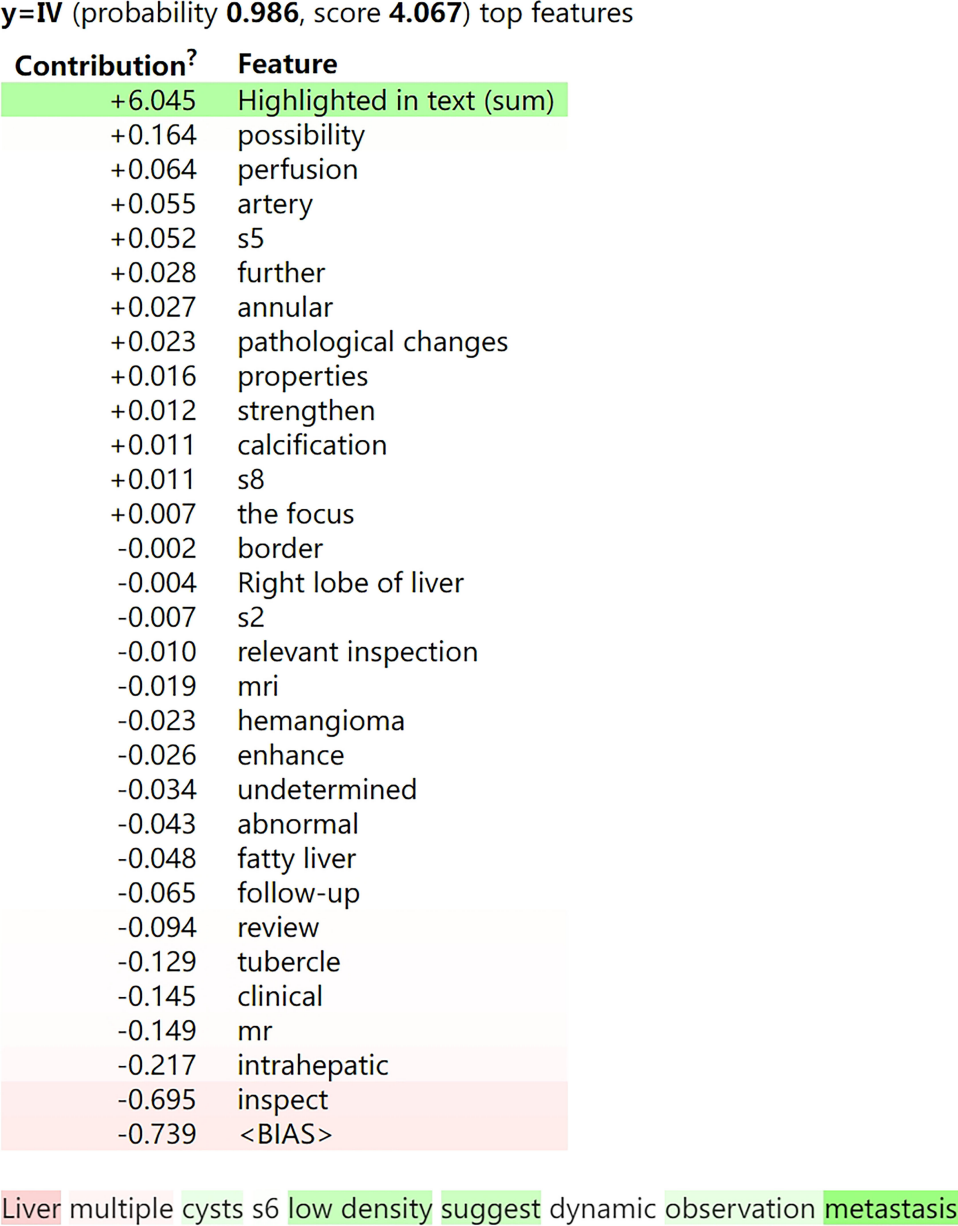
The classification result of an example of CT with/without contrast report. “y” was assigned to a certain category according to the probability, with green representing features that were positively related to this category, and the darker the green the greater the degree of correlation. Red represented features that were negatively related to this category, and the darker the red the less relevant they were. For publication purposes, we provided English version of Chinese words from the imaging reports.

We used ELI5 to select important feature words of the XGBoost classifier for use in the general classification, as shown in **Figure 5**. Green represented positive correlation, and the weight represented the contribution. The darker the green, the stronger the correlation between the word and classification. Words such as “no abnormality”, “suggest”, “fatty liver”, and “transfer” showed relatively large positive correlation. **Figure 6** showed an example of a radiological report classification. In this example, “y” was assigned to a certain category according to the probability, with green representing features that were positively related to this category, and the darker the green the greater the degree of correlation. Red represented features that were negatively related to this category, and the darker the red the less relevant they were. When “y” = IV, the probability reached 0.986 and the score was 4.067. Words such as “low density”, “suggest” and “metastasis” had a relatively large degree of positive correlation.

## Discussion

### Principal results

In this study, the NLP approach showed high accuracy in classification of liver medical imaging reports of patients with CRC according to clinical significance. These findings suggest that an ensemble learning classification model based on NLP could be an effective tool for determining the value of radiological reports focused on liver lesions. The advantages of our method were as follows: First, by applying this classification model, researchers can carry out large-scale clinical research in the future. For example, we can quickly establish a study group of CRC patients with/without liver metastasis from the imaging report. Secondly, classifying image reports according to clinical value can better understand the application of oncologists for liver examination of patients with CRC. This encourages oncologists to select appropriate examination, CT or MRI, in future clinical work. Thirdly, if this classification model is embedded in the medical record system in the future, clinicians can quickly know whether the patient has liver metastasis or other liver disease that need further management without reviewing the image report, so as to improve work efficiency.

The evaluation and management of distant metastases is generally very similar between colon and rectal cancer (21). Approximately 14.5% of patients with CRC present with synchronous liver involvement (22), although the great majority of patients with CRC undergo medical imaging examinations for pretreatment staging and detection of distant metastasis as a routine check. For the purpose of screening for liver metastasis, this study classified the medical imaging reports into five categories according to the degree of correlation with the examination purpose. Categories I and III excluded the diagnosis of liver metastasis. liver lesions of category III, such as primary carcinoma of the liver and lesions of unknown nature, should be further examined or treated by clinicians according to the patient’s condition. Category IV is suspected liver metastasis, and for diagnoses in this category, clinicians would need other examinations to make further confirmation. Category V is confirmed diagnosis of liver metastasis, and with this diagnosis clinicians could customize the treatment plan directly. With this classification result, clinicians could quickly respond to the medical imaging examination.

The study results revealed that most clinicians in our single center chose the imaging method of “CT with/without contrast” to screen for liver metastasis, which is consistent with the recommended methods in the clinical practice guidelines (23). Because of the long imaging time and high price, the use of MRI was not as high as that of CT. However, MRI showed higher detection ability than CT because of its high soft tissue resolution. Patients with a high suspicion of liver metastasis should be examined using MRI. Therefore, in our medical center, the largest proportion of liver metastases were confirmed by MRI, especially MRI with/without contrast.

### Comparison with prior work

Thus far, the primary focus of much of the research examining applications of NLP in the text analysis of medical imaging reports has been in serving health care providers (24–26). There are also some examples of NLP technology applied to clinical research. For instance, Kim et al. (27) classified brain MRI reports of acute ischemic stroke and non-acute ischemic stroke using NLP, and evaluated a variety of machine learning algorithms for this procedure, among which the F1 value (0.93) and accuracy (98.0%) of a single decision tree were the highest. Wheater et al. (28) developed a rule-based NLP algorithm to automatically identify brain MRI reports, and found sensitivity, positive predictive value, and specificity of 89%, 85%, and 100%, respectively, for identifying ischemic stroke reports, 96%, 72%, and 100% for identifying hemorrhagic stroke, and 96%, 84%, and 100% for recognizing brain tumors. Lee et al. (29) used NLP to infer the classification of brain tumor reports and a data system from unstructured brain MRI reports. They found that when classifying unstructured reports, section-wise ensemble models using XGBoost and word2vec semantic words were more accurate than a model using Tf-idf statistics, with an F1 value of 0.72. The model using traditional Tf-idf statistical data was better than the word2vec semantic method in structured report classification, with an F1 value of 0.98. There is great potential for applying these technologies in health care management. With increasing waiting times and patient lists in hospitals around the world, endpoints such as clinical value and resource utilization are becoming increasingly important for managers, providers, and patients.

In this study, we trialed the n-gram language model of NLP method. N-gram is an algorithm based on statistical language model. Its basic idea is that the contents of the text are operated by n-size sliding windows according to bytes, forming a sequence of n-length byte fragments. Each byte segment is called gram. The occurrence frequency of all grams is counted and filtered according to the preset threshold to form a list of key grams. N-gram language model shows good performance in many text mining tasks (30–32). For example, Giannakopoulos and Karkaletsis (30) expressed the text as an n-gram model using a sliding window with a length of n by connecting the adjacent n-grams with the edges representing their co-occurrence frequency in a given text window, they captured the word order in the text and detected some similarities in the text morphology.

Our study using NLP realized automatic classification of liver results from the text of medical imaging reports of patients with CRC. The classifier built from machine learning algorithms such as XGBoost and LR had extremely high sensitivity and specificity for the five classification categories applied to the liver radiological reports, and reached a high rate of accuracy. The classifier can strongly indicate the clinical value of the reports ordered by the clinician. The terms “no abnormality”, “clinical”, “re-examination”, “related examination”, and “metastasis” were among the important characteristics used for the classification. The confusion matrix revealed that categories III and IV were difficult to distinguish, which is similar to the experience of the radiologist. These two categories required further decision-making by doctors, which indicates that existing imaging methods might not play a definitive role in determining or excluding target lesions.

## Conclusion

The learning classification model based on NLP was an effective tool for determining the value of radiological reports focused on liver lesions. The study made it possible to analyze the value of medical imaging examinations on a large scale and it could be used to provide decision support for further clinical management of CRC patients in the future.

## Limitations

This preliminary study has several limitations. First, the classification model was trained on data from a single medical center, and the generalizability of the results is unknown. More work must be done to explore the application of the technology in different institutions and medical imaging research applications. Second, for most of the observations used to construct the dataset, our medical institution did not widely adopt a standardized framework for the description and classification of liver lesions. Standardized reporting using tools such as the Liver Imaging Reporting and Data System (33) can reduce the variability of reporting and improve model performance. However, despite the limitations of our dataset, the system operated with high accuracy in the classification. Third, we acknowledge that the common phrases in radiology literature can identify positive report of specific diseases without the need for artificial intelligence (AI). The purpose of our application of AI is not to identify a specific case, but to classify a large number of image reports according to clinical significance, so as to help understand the effectiveness of a certain imaging method for its clinical purpose. Finally, we have not embedded the report classification model into the medical record system because this is currently only preliminary research.

## Data availability statement

The raw data supporting the conclusions of this article will be made available by the authors, without undue reservation.

## Ethics statement

The studies involving human participants were reviewed and approved by Medical Ethics Review Board of Beijing Friendship Hospital Affiliated to Capital Medical University. The ethics committee waived the requirement of written informed consent for participation.

## Author contributions

This study was drafted by WL and XZ. ZW and HS took responsibility for all aspects of the research work to ensure that issues relating to the accuracy or integrity of any part of the paper were properly investigated and addressed. JL and HL did important contributions to the thinking of research work. WL obtained and analyzed the radiological medical reports for research. XZ was responsible for natural language processing of text. XW and YCL constructed different algorithm models. YWL and ZY finalized the version to be published. All authors contributed to the article and approved the submitted version.

## Funding

We thank the following fund projects for their support: No.[2015]160 from Beijing Scholars Program, No. yyqdktbh2020-9 from Beijing Friendship Hospital, Capital Medical University, No. 2021-135 from Beijing Key Clinical Discipline Funding and No. ZYLX202101 from Beijing Hospitals Authority Clinical Medicine Development of Special Funding Support. No. 2022-ZZ-001 from Beijing Postdoctoral Research Foundation, No. 82202258 from National Natural Science Foundation of China.

## Conflict of interest

The authors declare that the research was conducted in the absence of any commercial or financial relationships that could be construed as a potential conflict of interest.

## Publisher’s note

All claims expressed in this article are solely those of the authors and do not necessarily represent those of their affiliated organizations, or those of the publisher, the editors and the reviewers. Any product that may be evaluated in this article, or claim that may be made by its manufacturer, is not guaranteed or endorsed by the publisher.
